# Replication by the Epistasis Project of the interaction between the genes for IL-6 and IL-10 in the risk of Alzheimer's disease

**DOI:** 10.1186/1742-2094-6-22

**Published:** 2009-08-23

**Authors:** Onofre Combarros, Cornelia M van Duijn, Naomi Hammond, Olivia Belbin, Alejandro Arias-Vásquez, Mario Cortina-Borja, Michael G Lehmann, Yurii S Aulchenko, Maaike Schuur, Heike Kölsch, Reinhard Heun, Gordon K Wilcock, Kristelle Brown, Patrick G Kehoe, Rachel Harrison, Eliecer Coto, Victoria Alvarez, Panos Deloukas, Ignacio Mateo, Rhian Gwilliam, Kevin Morgan, Donald R Warden, A David Smith, Donald J Lehmann

**Affiliations:** 1 Neurology Service and Centro de Investigación Biomédica en Red sobre Enfermedades Neurodegenerativas (CIBERNED), Marqués de Valdecilla University Hospital (University of Cantabria), 39008 Santander, Spain; 2 Department of Epidemiology, Erasmus MC University Medical Center, Rotterdam, the Netherlands; 3 The Wellcome Trust Sanger Institute, Hinxton, Cambridge CB10 1SA, UK; 4 School of Molecular Medical Sciences, Institute of Genetics, University of Nottingham, Queens Medical Centre, Nottingham NG7 2UH, UK; 5 Centre for Paediatric Epidemiology and Biostatistics, Institute of Child Health, University College London, 30 Guilford Street, London WC1N 1EH, UK; 6 Oxford Project to Investigate Memory and Ageing (OPTIMA), University Department of Physiology, Anatomy and Genetics, South Parks Road, Oxford OX1 3QX, UK; 7 Department of Neurology, Erasmus MC University Medical Center, Rotterdam, the Netherlands; 8 Department of Psychiatry, University of Bonn, Bonn, Germany; 9 Department of Psychiatry, Derby City General Hospital, Uttoxeter Road, Derby DE22 3NE, UK; 10 Nuffield Department of Medicine, University of Oxford, Level 4, John Radcliffe Hospital, Oxford OX3 9DU, UK; 11 Dementia Research Group, Institute of Clinical Neurosciences, University of Bristol, Frenchay Hospital, Frenchay, Bristol BS32 8BE, UK; 12 Genética Molecular, Hospital Central de Asturias, Oviedo, Spain

## Abstract

**Background:**

Chronic inflammation is a characteristic of Alzheimer's disease (AD). An interaction associated with the risk of AD has been reported between polymorphisms in the regulatory regions of the genes for the pro-inflammatory cytokine, interleukin-6 (IL-6, gene: *IL6*), and the anti-inflammatory cytokine, interleukin-10 (IL-10, gene: *IL10*).

**Methods:**

We examined this interaction in the Epistasis Project, a collaboration of 7 AD research groups, contributing DNA samples from 1,757 cases of AD and 6,295 controls.

**Results:**

We replicated the interaction. For *IL6 *rs2069837 AA × *IL10 *rs1800871 CC, the synergy factor (*SF*) was 1.63 (95% confidence interval: 1.10–2.41, *p *= 0.01), controlling for centre, age, gender and apolipoprotein E ε4 (*APOE*ε4) genotype. Our results are consistent between North Europe (*SF *= 1.7, *p *= 0.03) and North Spain (*SF *= 2.0, *p *= 0.09). Further replication may require a meta-analysis. However, association due to linkage disequilibrium with other polymorphisms in the regulatory regions of these genes cannot be excluded.

**Conclusion:**

We suggest that dysregulation of both IL-6 and IL-10 in some elderly people, due in part to genetic variations in the two genes, contributes to the development of AD. Thus, inflammation facilitates the onset of sporadic AD.

## Background

Alzheimer's disease (AD) is accompanied by a chronic inflammatory process, including activation of microglia and astrocytes that express pro-inflammatory cytokines [1,2]. It is unclear to what extent this inflammation is a reaction to the pathology of AD, and to what extent it contributes to the onset or progression of the disease.

Two multi-functional cytokines, interleukin-6 (IL-6) and interleukin-10 (IL-10), may be relevant to this question. IL-6 is a potent pro-inflammatory cytokine [3], while IL-10 acts to limit inflammation in the brain [4]. Both are produced by activated microglia and astrocytes [3,4]. Two single nucleotide polymorphisms (SNPs), rs1800795 (-174G/C) and rs1800896 (-1082G/A), in the regulatory regions of the genes, *IL6 *and *IL10*, respectively, have been widely studied. However, the ongoing AlzGene meta-analyses of the two SNPs [5] are both currently negative (4 July 2009): pooled odds ratios for Caucasians in single-locus analyses of *IL6*-174C versus G alleles = 0.93 (95% confidence interval: 0.79–1.08, 14 studies) and of *IL10*-1082G versus A alleles = 0.91 (0.74–1.11, eight studies). Our own meta-analyses, both by allele and by genotype, confirmed these results and also indicated a high degree of heterogeneity among the studies (*p *< 0.05 in seven out of eight analyses, data not shown). Such inconsistencies may be due to study differences, e.g. in design or technical or analytical approach. However, the heterogeneity remained in six out of eight analyses after altogether four studies with controls in Hardy-Weinberg disequilibrium were removed. Alternatively, the heterogeneity may reflect true population differences, such as in interactions with other factors, including other genes. Such diverse results have been described as a marker of epistasis [6,7], i.e. where the effect of one polymorphism depends on the genotype at another locus.

Infante et al (2004) [8] reported an interaction between *IL6*-174G/C and *IL10*-1082G/A associated with the risk of AD. We therefore set out to replicate this result in the Epistasis Project.

### The Epistasis Project

Sporadic Alzheimer's disease (AD) is a complex disease, with over 50% heritability [9]. This suggests that its study requires the investigation of interactions between risk factors, particularly genetic factors. The Epistasis Project studies such interactions, mainly those between distinct genetic loci, i.e. epistasis. The project is a collaboration of seven AD research groups: Bonn, Bristol, Nottingham, OPTIMA, Oviedo, Rotterdam and Santander (Table 1). The project aims: first, to replicate genetic interactions that have been reported to affect the risk of sporadic AD; second, to explore other polymorphisms in the relevant genetic regions, ultimately to reveal the true risk loci. The overriding object is to gain insights into AD causality. However, interactions can only be reliably studied with sufficient statistical power and careful study design. The project therefore has these characteristics:-

**Table 1:** Sample-sets used in the Epistasis Project

Group	Geographical Region*	Subjects^†^	Numbers	% women	*p *(AD vs controls)	Median age^‡ ^(interquartile range)	*p *(AD vs controls)
Bonn	Bonn and Mainz, Germany	Clinical AD	259	61.4		71.0 (65.1–77.5)	
		Screened controls	232	56.9	0.31	67.8 (64.4–76.1)	0.11
Bristol	South-West England	Autopsy AD	200	45.0		80.4 (75.6–86.0)	
		Autopsy controls	57	40.4	0.55	77.9 (72.8–82.9)	0.12
Nottingham	Cambridge, England	Autopsy AD	104	48.1		78.7 (70.8–84.6)	
		Autopsy controls	107	34.6	0.051	72.5 (65.6–80.0)	< 0.001
OPTIMA	Oxford, England	Autopsy AD	163	54.6		79.5 (72.4–85.2)	
		Clinical AD	87	54.0		79.1 (73.4–84.8)	
		Screened controls	261	53.6	0.93	78.9 (72.9–83.8)	0.54
Oviedo	Asturias, Spain	Clinical AD	202	69.8		78.4 (74.8–82.8)	
		Screened controls	131	61.8	0.15	69.7 (63.5–74.5)	< 0.001
Rotterdam	Rotterdam (Ommord), the Netherlands	Clinical AD	391	74.9		86.6 (82.2–91.6)	
		Screened controls	5,111	57.7	< 0.001	76.9 (71.7–83.0)	< 0.001
Santander	Cantabria, Spain	Clinical AD	351	65.0		75.5 (70.8–78.9)	
		Screened controls	396	68.9	0.27	80.9 (74.7–85.6)	< 0.001

Totals		Total AD	1,757	62.4		79.0 (73.0–85.2)	
		Total controls	6,295	57.7		76.9 (71.3–83.0)	

AD = Alzheimer's disease

*from which the samples were drawn.

^†^Clinical AD cases were diagnosed as "probable AD" by NINCDS-ADRDA criteria [12]; autopsy AD cases were diagnosed as "definite" or "probable" by CERAD criteria [11]; screened controls were free of cognitive impairment; autopsy controls were free of pathology consistent with AD or other dementias; all controls were ≥ 60 years of age.

^‡^Age at death or last examination.

#### 1. Power

The project has 1,757 cases of AD and 6,295 elderly controls. These numbers give 99.9% power to detect an *SF *[10] of 2 between two polymorphisms, each with a minor allele frequency of 20%, when controlling for individual centres (2 below). They give 89.8% power or 48.6% power to detect *SF*s of 1.5 or 1.25, respectively. Quality control of genotyping reduces the numbers somewhat, depending on the polymorphism.

#### 2. Selection of sample-sets

Only sample-sets drawn from narrow geographical regions with relatively homogeneous, Caucasian populations have been chosen, from seven AD research centres (Table 1 and Additional file 1).

#### 3. Sample characterisation

Cases of AD are either confirmed at autopsy as "definite" or "probable" by CERAD criteria [11], or clinically diagnosed as "probable AD" by NINCDS-ADRDA criteria [12]. Controls are either screened as free of cognitive impairment, or confirmed at autopsy as free of pathology consistent with AD or other dementias.

#### 4. Matching of cases and controls

Only cases and controls drawn from the same region are compared, thus controlling for geographical differences, e.g. between North and South Europe.

#### 5. Candidate interactions

Interactions are studied that have prior evidence of an association with AD and a plausible biological hypothesis. Interactions with age (± 75 years), gender and apolipoprotein E ε4 (*APOE*ε4) genotype are also examined.

#### 6. Analytical methods

Logistic regression and *SF *[10] analyses are used. All analyses are controlled for age, gender and *APOE*ε4 genotype. All pooled analyses are also controlled for centre. There is thus no question of controls from one centre (e.g., Rotterdam) being compared with AD cases from another region. Controlling for centre also reduces the relative influence of large subgroups, such as Rotterdam controls, in the pooled result. Where pooled analyses yield significant results, the seven individual centres are also examined for associations with AD, for heterogeneity, and for power to detect the interaction.

Table 1 gives the basic characteristics of the seven sample-sets. See Additional file 1 for further information.

To select the interactions for study, a survey of over 100 published claims and suggestions of epistasis in sporadic AD was undertaken [13]. The interactions finally chosen are involved in various networks that are widely considered to contribute to the development of AD: lipid metabolism [14], β-amyloid metabolism [15], oxidative stress [16], inflammation [1], insulin metabolism [17] and homocysteine metabolism [18]. Positive results should therefore deliver insights into the causes of AD.

## Methods

Basic information on the 1,757 cases of AD and the 6,295 controls from the seven centres is given above and in Table 1, and fuller details are provided in Additional file 1.

Genotyping for the six centres other than Rotterdam was performed at the Wellcome Trust Sanger Institute, using the iPLEX Gold assay (Sequenom Inc.). Whole genome amplified DNA was used for 82% of samples; genomic DNA was used for the 18% of samples that were not suitable for whole genome amplification. A Sequenom iPLEX, designed for quality control purposes, was used to assess genotype concordance between genomic and whole genome amplified DNA for 168 individuals. Assays for all SNPs were designed using the eXTEND suite and MassARRAY Assay Design software version 3.1 (Sequenom Inc.). Samples were amplified in multiplexed PCR reactions before allele specific extension. Allelic discrimination was obtained by analysis with a MassARRAY Analyzer Compact mass spectrometer. Genotypes were automatically assigned and manually confirmed using MassArray TyperAnalyzer software version 4.0 (Sequenom Inc.). Gender markers were included in all iPLEX assays as a quality control metric for confirmation of plate/sample identity. Genotyping of SNPs rs1800871, rs2069837 and rs3024505 was carried out using the KASPar technology by KBioscience .

Genotyping in the Rotterdam cohort was done on Version 3 Illumina-Infinium-II HumanHap550 SNP array (Illumina, San Diego, USA) and additionally, SNPs were imputed using MACH software  with HapMap CEU Release 22 as a reference [19]. The reliability of imputation was estimated for each imputed SNP with the ratio of expected and observed dosage variance (O/E ratio). Only samples with high-quality extracted DNA were genotyped; 5974 were available with good quality genotyping data; 5502 of these had reliable phenotypes. For the Epistasis Project, 52 genotyped SNPs and 116 imputed SNPs were selected.

We assessed associations with logistic regression models and *SF *analysis [10], controlling for age, gender, *APOE*ε4 and study centre, using R Version 2.6.1 (R Foundation for Statistical Computing, Vienna, Austria). Heterogeneity between centres was controlled by fitting a fixed effect corresponding to contrasts between the baseline centre and the six other centres (having compared models with fixed- and random-effect terms in centre, goodness of fit was measured using Akaike's Information Criterion, which favoured using fixed effects only). Where the overall *SF *was significant at p < 0.05, the seven individual centres and the two geographical regions, North Europe and North Spain, were also examined. Power calculations were by *SF *analysis. Meta-analyses were performed using the random-effect method of DerSimonian and Laird [20] and the heterogeneity test of Armitage [21]. Comparisons of allelic frequencies between North Spain and North Europe were by Fisher's exact test. We compared the medians of the age distributions of AD and control groups using the Wilcoxon-Mann-Whitney test. Linkage disequilibrium data were estimated using the R genetics library . All tests of significance were two-sided.

## Results

We replicated the interaction reported by Infante et al (2004) [8] between *IL6 *rs1800795 (-174G/C) and *IL10 *rs1800896 (-1082G/A) (below). In the Rotterdam cohort, we examined altogether five *IL6*-related SNPs, rs1800797, rs1800795, rs2069837, rs2069840 and rs2069845, and four *IL10*-related SNPs, rs1800896, rs1800871, rs3024498 and rs3024505. In that preliminary study, we found that the strongest interactions were seen between *IL6 *rs2069837 (intron 2 A/G) and *IL10 *rs1800871 (-819C/T) and between *IL6 *rs2069837 and *IL10 *rs3024505 (3' C/T) (data not shown). We therefore studied the five SNPs shown in Table 2 in all seven sample-sets.

**Table 2:** Studied SNPs

Gene	SNP		Minor allele frequency in controls			LD in controls		
			
			North Europe	North Spain	Difference (*p*)	With	D'	r^2^
*IL6*	rs1800795	-174G/C	41.1% (C)	32.8% (C)	< 0.0001	rs2069837	0.998	0.055
	rs2069837	Intron 2 A/G	7.3% (G)	9.3% (G)	0.03			

*IL10*	rs1800896	-1082G/A	50.7% (G)	43.1% (G)	< 0.0001	rs1800871	0.999	0.296
						rs3024505	0.988	0.191
	rs1800871	-819C/T	22.5% (T)	26.7% (T)	0.002	rs3024505	0.999	0.058
	rs3024505	3' C/T	16.6% (T)	14.2% (T)	0.06			

SNP = single nucleotide polymorphism; LD = linkage disequilibrium; D' = ratio of observed LD to maximum possible LD; r = correlation coefficient; *IL6 *and *IL10 *= the genes for interleukin-6 and interleukin-10, respectively.

Hardy-Weinberg analysis was performed for the five SNPs of Table 2 in both cases and controls of the Rotterdam samples, genotyped by Rotterdam, and of the samples from the other six centres, genotyped by the Sanger Institute. As expected by chance, one of these 20 analyses resulted in disequilibrium: *IL6*-174G/C in AD cases of the six centres (*p *= 0.02). The two *IL6 *SNPs were in linkage disequilibrium (LD), as were the three *IL10 *SNPs (Table 2). The allelic frequencies differed significantly between North Europe (Bonn, Bristol, Nottingham, OPTIMA and Rotterdam) and North Spain (Oviedo and Santander) in four out of the five SNPs (Table 2).

We examined the potential associations with AD of the six interactions generated between the two *IL6 *SNPs and the three *IL10 *SNPs in our overall dataset (Table 3), controlling for centre, age, gender and *APOE*ε4 genotype (as in all association analyses). Three of the six interactions were associated with AD: *SF p *< 0.05. The first interaction shown in Table 3 is effectively identical to that reported by Infante et al 2004 [8]; we have merely reversed the first genotype, i.e. CC rather than GC + GG, to give an *SF *of 1.56, rather than its inverse, 0.64, for easier comparison with the other interactions. The two *IL6 *genotypes shown in Table 3 were in LD (*p *< 0.0001), as were the three *IL10 *genotypes (*p *< 0.0001). The interaction between *IL6 *intron 2 AA and *IL10*-819CC was slightly the strongest: *SF *= 1.63 (95% confidence interval = 1.10–2.41, *p *= 0.01). All further analysis was therefore restricted to that interaction.

**Table 3:** Potential interactions between *IL6 *SNPs and *IL10 *SNPs in the risk of AD

Interaction		Numbers		Adjusted* synergy factor(95% CI, *p*)
	
*IL6*	× *IL10*	Controls	AD cases	
rs1800795 CC	× rs1800896 G+	6153	1557	**1.56 (1.02–2.38, 0.04)**
rs1800795 CC	× rs1800871 CC	6158	1514	1.25 (0.86–1.80, 0.25)
rs1800795 CC	× rs3024505 T+	6190	1546	1.11 (0.75–1.64, 0.60)
rs2069837 AA	× rs1800896 G+	6126	1437	1.37 (0.89–2.11, 0.16)
rs2069837 AA	× rs1800871 CC	6150	1442	**1.63 (1.10–2.41, 0.01)**
rs2069837 AA	× rs3024505 T+	6172	1429	**1.55 (1.02–2.36, 0.04)**

*IL6 *and *IL10 *= the genes for interleukin-6 and interleukin-10, respectively; SNPs = single nucleotide polymorphisms; AD = Alzheimer's disease; CI = confidence interval; G+ and T+ group the genotypes, GA+GG and CT+TT, respectively; *APOE*ε4 = apolipoprotein E ε4.

*All analyses controlled for centre, age, gender and *APOE*ε4 genotype.

Table 4 shows the effect of each of the two factors in that interaction on the association with AD of the other factor. The presence of *IL10*-819CC changed the association of *IL6 *intron 2 AA with AD from negative (odds ratio = 0.86, *p *= 0.30) to risk (odds ratio = 1.38, *p *= 0.02), while the presence of the latter removed the protective association of the former (odds ratio = 0.63, *p *= 0.01, changed to 1.08, *p *= 0.32).

**Table 4:** Odds ratios of Alzheimer's disease for two *IL6 *and *IL10 *SNPs, stratified by each other

Odds ratio of AD for :-	In the subset :-	Numbers		Adjusted* odds ratio of AD (95% CI, *p*)
			
		Controls	AD cases	
*IL6*	*IL10*	AA: 2158	AA: 518	0.86 (0.64–1.15, 0.30)
rs2069837	rs1800871 T+	G+: 348	G+: 87	
AA vs G+	*IL10*	AA: 3099	AA: 724	**1.38 (1.06–1.79, 0.02)**
	rs1800871 CC	G+: 545	G+: 113	

*IL10*	*IL6*	CC: 545	CC: 113	**0.63 (0.44–0.91, 0.01)**
rs1800871	rs2069837 G+	T+: 348	T+: 87	
CC vs T+	*IL6*	CC: 3099	CC: 724	1.08 (0.93–1.25, 0.32)
	rs2069837 AA	T+: 2158	T+: 518	

*IL6 *and *IL10 *= the genes for interleukin-6 and interleukin-10, respectively; AD = Alzheimer's disease; CI = confidence interval; G+ and T+ group the genotypes, GA+GG and CT+TT, respectively; *APOE*ε4 = apolipoprotein E ε4.

*All analyses controlled for centre, age, gender and *APOE*ε4 genotype.

There was significant heterogeneity between the centres, and the interaction was only significant in one of the seven centres, Rotterdam, with an *SF *of 3.0 (95% confidence interval = 1.6–5.8, *p *= 0.0007). But the power to detect an *SF *of 1.6 was low in each centre, ranging from below 10% to 40%. The results for North Europe (*SF *= 1.7, *p *= 0.03) and for North Spain (*SF *= 2.0, p = 0.09) were consistent (Table 5).

**Table 5:** Interaction between *IL6 *rs2069837 AA vs G+ and *IL10 *rs1800871 CC vs T+, by region

Region	Power*	Adjusted^† ^synergy factor (95% CI, *p*) in the risk of AD
North Europe^‡^	64%	**1.7 (1.05–2.6, 0.03)**
North Spain^‡^	37%	2.0 (0.9–4.4, 0.09)
All	76%	**1.6 (1.1–2.4, 0.01)**

*IL6 *and *IL10 *= the genes for interleukin-6 and interleukin-10, respectively; G+ and T+ group the genotypes, GA+GG and CT+TT, respectively; CI = confidence interval; AD = Alzheimer's disease; *APOE*ε4 = apolipoprotein E ε4.

*To detect a synergy factor of 1.6 (as in the overall dataset) at 0.05.

^†^All analyses controlled for centre, age, gender and *APOE*ε4 genotype.

^‡^North Europe = Rotterdam, Bonn, OPTIMA, Nottingham and Bristol; North Spain = Santander and Oviedo.

Results were similar for men as for women (data not shown). The interaction was slightly stronger in older subjects: *SF *for > 75 years = 1.71 (1.05–2.78, 0.03); *SF *for < 75 years = 1.27 (0.56–2.87, 0.57). However, there was no 3-way interaction with age. Nor did we find a significant interaction with *APOE*ε4.

## Discussion

In our whole dataset of over 7,500 samples, we have replicated both the interaction reported by Infante et al 2004 [8] and also that found in our preliminary study in the Rotterdam dataset, between SNPs of *IL6 *and *IL10 *(Table 3). Both interactions gave significant *SF*s of approximately 1.6. There was significant heterogeneity between centres, which was unsurprising since only two, Rotterdam and Santander, had > 20% power to detect these interactions. But the results for the two main regions, North Europe and North Spain, were consistent (Table 5). We conclude that this is likely to be a true effect, but not a very strong one. As in any example of true epistasis, the presence or absence of one factor critically influenced the effect of the other (Table 4).

Further replication will require a dataset at least as large, with appropriate statistical control for differences between individual sample-sets. Underpowered studies are unhelpful, since chance can produce misleading results in such cases. This criticism may apply to a previous study of this interaction that had power of < 2% to detect it. Even the large Rotterdam Study, with 395 cases of AD and 5,111 controls, only had 40% power to detect this interaction. Thus, further replication may require a meta-analysis.

### IL-6 in age-related decline and in AD

There is much evidence that chronic, low-grade overexpression of IL-6 contributes to age-related decline. Blood levels of IL-6 can rise with age in humans [22-24]. Raised levels are associated with various age-related conditions, including risk of cognitive decline in some studies [25,26], but not in all [27]. Raised levels have been consistently associated with increased mortality in several large prospective studies [28-31]. Raised levels have also been associated with various conditions considered to be risk factors for dementia and/or AD: subclinical and clinical cardiovascular disease and the risk thereof [32-34], type 2 diabetes and its risk [35,36], psychological stress [37] and the damage following stroke (IL-6 also increased in CSF) [38,39].

Further, raised blood levels of IL-6 have often been associated with AD [40-42] and also with the risk of AD [43], although not in all studies [44,45]. Post-mortem studies, although small, have generally reported increased IL-6 levels or changed IL-6 distribution in AD brain [46-49], with one exception [50]. IL-6 was found not only in plaques, but also around the bodies of isocortical neurones, only in AD [46]. This evidence and that above suggest that dysregulation of IL-6 contributes to the development of AD.

### A potential interaction between *IL6* and *IL10*

Both IL-6 and IL-10 are produced by activated microglia and astrocytes (reviewed in [3,4]). However, in contrast to IL-6, IL-10 acts to limit inflammation in the brain. IL-10 inhibits the production of IL-6 [51,52] and its receptor [53]. Thus, certain combinations of genetic variants of *IL6 *and *IL10*, i.e. those associated with high production of IL-6 combined with those associated with low production of IL-10, may contribute to the dysregulation of inflammation. High heritability (> 50%) has been reported for *in vitro *stimulated production of both IL-6 and IL-10 [54]. The functions of two of our studied SNPs, *IL6 *intron 2 A/G and *IL10 *3' C/T have not yet been investigated. Thus we cannot rule out that they might have effects on transcription or post-transcriptional processing. Alternatively, they may be in linkage disequilibrium with other, functional variants. On the other hand, studies of *IL6*-174G/C [55,56], of *IL10*-819C/T [57] and of *IL10*-1082G/A [58-60] have reported that these SNPs affect transcription or are in linkage disequilibrium with those that do. However, it may be premature to designate any particular alleles as high or low producers, since there have been contrasting results for the effects of *IL6*-174G/C and *IL10*-1082G/A on transcription, on *in vitro *stimulated production and on blood levels of the respective proteins. But most studies have associated the *IL10*-1082GG genotype at least with higher *in vitro *stimulated production of IL-10 [61-64], although not all, [65,66] and results have varied with experimental conditions [64,66]. It appears that several SNPs in the regulatory regions of each gene affect transcription in an interactive and tissue-specific manner [56]. Indeed, we note that in our dataset the 2-SNP combination of *IL10 -1082G+/-819CC* has a slightly stronger interaction with *IL6 *than that of either SNP alone (data not shown).

## Conclusion

We conclude that an interaction between IL-6 and IL-10 is plausible; that dysregulation of the two genes contributes to chronic low-grade inflammation in some elderly people and thus to the risk of AD; and that certain combinations of genetic variants in the regulatory regions of the two genes are conducive to this dysregulation. But in view of the linkage disequilibrium in the region of each gene (Table 2), we cannot conclude that we have yet found the true risk polymorphisms. However, we suggest that our results are consistent with the contribution of inflammation to the onset of AD.

## Abbreviations

AD: Alzheimer's disease; *APOE*ε4: apolipoprotein E ε4; CERAD: Consortium to Establish a Registry for Alzheimer's Disease; CSF: cerebrospinal fluid; IL-6: interleukin-6; *IL6*: the gene for interleukin-6; IL-10: interleukin-10; *IL10*: the gene for interleukin-10; NINCDS-ADRDA: National Institute of Neurological, Communicative Diseases and Stroke-Alzheimer's Disease and Related Diseases Association; OPTIMA: the Oxford Project to Investigate Memory and Ageing; *SF*: synergy factor; SNP: single nucleotide polymorphism.

## Competing interests

The authors declare that they have no competing interests.

## Authors' contributions

All authors contributed to the design of the study. In addition, ADS and DJL set up the Epistasis Project, with the help of the other authors. ADS and DJL decided on the strategy of the Epistasis Project, with the help of CMvD, OC, KM, PK, R Heun, MC-B, DRW and EC. ADS, DJL, CMvD, OC, KM, PK, R Heun, MC-B, DRW and EC chose the genetic interactions to study. OC produced the hypothesis for this study. KM and OB gave extensive advice on the choice of SNPs to study. DJL made the final selection of polymorphisms. HK, R Harrison, KM, DRW, EC and IM provided DNA for genotyping. CMvD, YSA, AA-V and MS provided the data for the preliminary study. DRW gave technical advice throughout. RG and NH were responsible for the genotyping of 6 sample-sets. AA-V was responsible for the Rotterdam genotyping. MC-B and DJL decided on the analytical approach. MC-B and YSA advised on statistics throughout. DJL and MGL performed the analysis. DJL drafted the manuscript. OC submitted the manuscript and is responsible for correspondence. All authors read the manuscript, studied it critically for its intellectual content and approved the final draft.

## Supplementary Material

Additional file 1 **The seven centres of the Epistasis Project**. Click here for file
