# Supplementary material for: Replication by the Epistasis Project of the interaction between the genes for IL-6 and IL-10 in the risk of Alzheimer's disease
Source: J Neuroinflammation. 2009 Aug 23;6:22. doi: 10.1186/1742-2094-6-22 (PMC2744667; doi:10.1186/1742-2094-6-22)
Supplement: Additional file 1 — The seven centres of the Epistasis Project. [file 1742-2094-6-22-S1.doc]

**The seven centres of the Epistasis Project**

**Bonn**

AD patients were recruited from the Department of Psychiatry, University of Bonn, Germany. Patients were diagnosed according to DSM-IV, supported by clinical examination, detailed structured interviews, neuropsychological testing, cognitive screening including Mini Mental State Examination (MMSE). Healthy controls were recruited with the support of the local Census Bureau and the regional Board of Data Protection (Nordrhein-Westfalen, Germany). The cognitive status was assessed by neuropsychological testing and structured interviews. All participants of the study gave informed written consent. The study has been approved by the Ethics Committee of the Faculty of Medicine of the University of Bonn, Germany.

**Bristol**

The University of Bristol is home to the South West Dementia Brain Bank (Human Tissue Authority licence number 12273) from which DNA from 200 AD patients and 57 controls were used in the Epistasis Project with local Research Ethics Committee approval. Of the patient group which had a mean age at death of 80.6(±8.4) years, 46% (n=90) were female. The control group with a mean age at death of 79.0(±8.0) years were 40.4% (n=23) female. All AD patients were sporadic AD whereby patients with any strong autosomal dominant family history of AD were excluded. All patient and controls were histopathologically confirmed by post-mortem autopsy according to the criteria of the Consortium to Establish a Registry for Alzheimer's Disease (CERAD) and controls were defined by the absence of AD or other neuropathological abnormalities.

**Nottingham**

The University of Nottingham is the coordinating centre for the Alzheimer’s Research Trust DNA Bank which contains 4000 samples from 8 UK centres. The Epistasis Project utilized 211 samples from this resource that originated from Cambridge, England. All subjects gave informed consent to be included in the study, which was granted by the local Ethics Committee. Of the 211 samples analysed 107 were controls (34.6% female, n=37) with a mean age of 73.4+/- 8.6 and 104 were AD (48.1% female, n=50) with a mean age of onset of 78.2+/- 8.6. Samples were histopathologically confirmed as definite AD or control using CERAD criteria (Mirra et al, 1991). All patients with evidence of an autosomal AD trait, or where a first degree relative had been diagnosed with familial AD, were excluded.

**OPTIMA**

The Oxford Project to Investigate Memory and Ageing (OPTIMA) is a longitudinal clinico-pathological study that began in 1988 (for details and references see: <http://www.medsci.ox.ac.uk/optima)>. By 2009, more than 1,200 subjects have been included in the project.

Patients with varying degrees of cognitive dysfunction were recruited from general practices and hospital clinics in the Oxfordshire Health Authority area in England. At the same time, elderly volunteer controls without symptoms of memory impairment were recruited by leaflets or by lectures given at retirement association clubs or from general practices in the same area

during the same period. All subjects underwent a detailed clinical history, physical examination, assessment of cognitive function (Cambridge Examination for Mental Disorders of the Elderly [CAMDEX], from which the Cambridge Cognitive Examination [CAMCOG] and Mini-Mental State Examination [MMSE] scores were derived) annually. A clinical diagnosis of possible or probable Alzheimer's disease was made using the NINCDS-ADRDA criteria. X-ray cranial computed tomography scans were performed annually using both the

standard axial angle and the temporal lobe-oriented angle. The minimum thickness of the medial temporal lobe at the level of the brainstem was measured from hard copies of the temporal lobe-oriented scan by two independent observers who were unaware of the diagnosis and previous scans. Single-photon computed tomography was performed on all subjects, using

CERETEC to reveal regional blood flow patterns. Non-fasting blood samples (serum and EDTA plasma) were taken at first visit and stored at -70°C. Whole blood and the buffy coat was also stored for future extraction of DNA.

All the above investigations were repeated annually and subjects who died were referred to the pathologist for histopathological study of the brain. A diagnosis of probable or definite Alzheimer's disease was established using the CERAD criteria.

**Oviedo**

The study included 202 patients (141 women; mean age 79 *±* 6 years) who fulfilled the National Institutes of Neurological and Communicative Diseases and Stroke-Alzheimer Disease

and Related Disorders (NINCDS-ADRDA) criteria for clinical probable AD. A total of 131 healthy elderly controls (mean age 70 *±* 7 years; 81 women) were also genotyped. They

were from the general population or healthy spouses of the patients. All the patients and controls were Spanish Caucasians and from the same region (Asturias, Northern Spain, total population one million), and gave their informed consent to participate in the study. This research was approved by the Ethical Committee of Hospital Central Asturias.

**The Rotterdam Study**

The Rotterdam Study is a prospective population-based cohort-study, which was designed for studying prevalence and risk factors for chronic diseases in the elderly (age over 55 years) [1]. At baseline (1990-1993) and follow-up, participants were invited for extensive examinations; all participants gave written informed consent.

Alzheimer’s disease (AD) was diagnosed with a three-step protocol [2]. Participants were cognitively tested during their visits to the research centre and screened with the Mini Mental State Examination (MMSE) and Geriatric Mental State schedule (GMS). Individuals with MMSE < 26 or on GMS > 0 were further tested with the Cambridge examination for mental disorders of the elderly (Camdex). When additional testing was required, participants were examined by a neuropsychologist and imaging data was used when available. The final diagnosis was ascertained by an expert team, consisting of a neurologist, neuropsychologist and research physician, according to internationally accepted criteria. Additionally, the population was continuously monitored for AD through medical records of general practitioners and the Regional Institute for Outpatient Mental Health Care. For the current study, 391 AD patients (incident AD) and 5111 controls were included with good quality data on genotypes and phenotypes.

**1. Hofman A, Breteler MM, van Duijn CM, Krestin GP, Pols HA, Stricker BH, Tiemeier H, Uitterlinden AG, Vingerling JR, Witteman JC: The Rotterdam Study: objectives and design update. *Eur J Epidemiol* 2007, 22(11):819-829.**

**2. Ott A, Breteler MM, van Harskamp F, Stijnen T, Hofman A: Incidence and risk of dementia. The Rotterdam Study. *Am J Epidemiol* 1998, 147:574-580.**

**Santander**

The study included 351 AD patients (65% women; 49 subjects with early-onset or age at onset < 65 years and 302 subjects with late-onset or age at onset ≥ 65 years; mean age

at study 75.3 years; S.D. 7.7; range 50–97 years; mean age at onset 72.2 years; S.D. 7.8; range 48–93 years) who met NINCDS/ADRDA criteria for probable AD. All AD cases were defined as sporadic because their family history did not mention any first-degree relative with dementia. AD patients were admitted to the Department of Neurology, University Hospital “Marqués de Valdecilla”, Santander, Spain, from January, 1997, to December 2001. The large majority of patients were living in the community and had been referred by their general practitioner; a few had been admitted from hospital wards or nursing home facilities. Control subjects were 396 unrelated individuals (69% women; 6 subjects with age at sampling < 65 years and 436 subjects with age at sampling ≥65 years; mean age 80.9 years; S.D. 7.8; range 51–100 years), randomly selected from a nursing home. These subjects had complete neurologic and medical examinations that showed that they were free of significant illness and had Mini Mental State Examination scores of 28 or more, which were verified by at least one subsequent annual follow-up assessment. The controls arose from the same base population as the cases. The AD and control samples were Caucasians originating from a limited geographical area in Northern Spain. All patients and controls were ascertained to have parents and grandparents born in Northern Spain to ensure ethnicity. Consequently, possible confounding effects of the inclusion in the study of members of different ethnic groups have been minimised

Combarros2009,Additional file,7Jly'09
